# Pandemic (H1N1) 2009 influenza virus induces weaker host immune responses *in vitro*: a possible mechanism of high transmissibility

**DOI:** 10.1186/1743-422X-8-140

**Published:** 2011-03-25

**Authors:** Sanjay Mukherjee, Veena C Vipat, Akhilesh C Mishra, Shailesh D Pawar, Alok K Chakrabarti

**Affiliations:** 1 Microbial Containment Complex, National Institute of Virology, Sus Road, Pashan, Pune -411021, India

## Abstract

**Background:**

The world has recently overcome the first influenza pandemic of the 21st century caused by a novel H1N1 virus (pH1N1) which is a triple reassortant comprising genes derived from avian, human, and swine influenza viruses and antigenically quite different from seasonal H1N1 strains. Although the case fatality rates have decreased in many developed countries, the situation is still alarming in many developing countries including India where considerable numbers of new cases are appearing everyday. There is still a high morbidity and mortality of susceptible adult as well as young population without having underlying health issues due to the influenza infection.

**Results:**

To achieve a better understanding of the risk posed by the pH1N1 and to understand its pathogenicity, we studied the host gene expression response to Indian isolate of pH1N1 infection and compared it with seasonal H1N1 infection. The response was studied at four different time points (4, 8, 16 and 24 h) post infection (hpi) in A549 cells using microarray platform. We found that pH1N1 induces immune response earlier than seasonal H1N1 viruses, but at the later stages of infection there is a suppression of host immune responses. The infection with pH1N1 resulted in considerable decrease in the expression of cytokine and other immune genes namely IL8, STAT1, B2 M and IL4 compared to seasonal H1N1.

**Conclusion:**

We propose that the inability to induce strong innate immune response could be a reason for the high transmissibility, pathogenicity and mortality caused by pH1N1 virus.

## Background

The pandemic (H1N1) 2009 influenza A virus (pH1N1) has already killed more than 19,000 people worldwide since it appeared in April 2009 [1]. Although on 10^th ^of August 2010, the Director General of the World Health Organization (WHO) has announced that the world is no longer in phase 6 of influenza pandemic alert and we are now moving into the post-pandemic period, the virus transmission is still highly active in many parts of South Asia, West Africa, and Central America [2]. In Asia, the most active areas of pandemic influenza virus transmission currently are in parts of India, Bangladesh, Bhutan, Myanmar Nepal, and Thailand. The virus (pH1N1) is still a serious threat to children as well as susceptible young and old population in developing countries like India.

Till date, there are reports of 2720 deaths from pandemic H1N1 influenza virus infection in India which is approximately 14% of the total world mortality http://mohfw-h1n1.nic.in/august.html;http://netindian.in/news/2010/11/15/0008699/6-h1n1-deaths-india-during-past-week-govt. The pandemic H1N1 virus is antigenically distinct from seasonal influenza viruses and the majority of human population lacks immunity against this virus [3-5]. The pathogenesis and transmission of the pH1N1 in humans is not completely known and many studies are underway. Animal studies have shown that this virus has a higher replicative power than the seasonal influenza virus [6,7]. Studies on human macrophages have shown that pH1N1 is a weak inducer of cytokine responses as compared to seasonal H1N1 viruses [8]. In addition, pH1N1 replicates efficiently in non-human primates, causes more severe pathological lesions in the lungs than currently circulating seasonal human H1N1 virus [9,10]. Earlier findings indicate that pandemic H1N1 are more pathogenic in mammalian models than seasonal H1N1 influenza viruses [6-8,11-13]. The pH1N1 isolates tested in mice and ferrets, were found to be replicating more efficiently than currently circulating human H1N1 viruses [6-8].

The respiratory tract is the primary site of infection for all the mammalian influenza viruses [13,14]. In this study we have used human lung epithelial cells (A549) to study the host gene expression responses to infection with Indian isolate of pH1N1, isolated in August 2009 and compared it with seasonal H1N1 infection in order to assess the pathogenicity and transmissibility of pandemic (2009) H1N1 influenza virus.

## Materials and methods

### Viruses and cell line

Pandemic Influenza virus A/Jalna/NIV9436/2009 (H1N1) and seasonal human influenza virus A/NIV/0914864/2009(H1N1) isolated in the influenza division of the National Institute of Virology, Pune, India were used for the study. Human lung epithelial (A549) cell line was used as host and maintained in Dulbecco's modified Eagle's tissue culture medium (Invitrogen Life Technologies, Carlsbad, CA, USA) containing 10% fetal calf serum,100 units/ml penicillin, 100 ug/ml streptomycin in tissue culture flasks (Corning, USA) at 37°C in a CO_2 _incubator.

### Virus infection

A549 cells were infected with pH1N1 and seasonal influenza viruses at a multiplicity of infection (MOI) of 3 as described earlier [15]. After 1 hour of adsorption period, the inoculum was removed and the cells were washed twice with phosphate buffer saline (PBS) and supplemented with growth media. Four sets of tissue culture flasks containing monolayer of A549 cells were infected corresponding to four different time points post infection for both the viruses. Mock infected cells at each time point served as controls. Infection of pH1N1 was performed in BSL-2 laboratory following World Health Organization norm for handling of pandemic H1N1 viruses.

### Microarray Hybridization

Infected cells were harvested at different time points post infection, total RNA was extracted from the infected cells at 4, 8, 16 and 24 hpi using Trizol reagent (Invitrogen Life Technologies, Carlsbad, CA, USA) and purified using the RNeasy kit (Qiagen, Germany). Amplification of RNA and indirect labeling of Cy-dye was done using Amino Allyl MessageAmp II aRNA amplification kit (Ambion, Austin, TX, USA) following manufacturer's instruction. One hundred nanograms of total RNA from control and infected cells were used for the experiments. The RNA was reverse transcribed and amplified according to manufacturer's protocol. The purified amino allyl aRNA was labeled with Cy3 and Cy5 for control and experimental samples respectively. Purified samples were lyophilized, resuspended in hybridization buffer (Pronto Universal Hybridization kit, Corning) and hybridized on human Discover chip (Arrayit corporation, Sunnyvale, CA, USA). Hybridization was carried out in a Hybstation (Genomic Solutions, Ann Arbor, MI) and the conditions used were 55°C for 6 h, 50°C for 6 h, and 42°C for 6 h. Scanning was performed at 5-micron resolutions with the Scan array express (PerkinElmer, Waltham, MI). Grid alignment was done by gene annotation files and raw data were extracted into MS EXCEL [15].

### Data Analysis

Microarray data analysis was carried out with GENOWIZ Microarray data and pathway analysis tool (Ocimum Biosolutions, Hyderabad, India). Replicated values for genes were merged and median values of the expression ratios were considered for the dataset. Undetected spots were removed by filtering. Dye bias was nullified by applying Loess normalization. Log transformation (log2) was done to stabilize the variation in dataset and median centering was performed to bring down data distribution of dataset close to zero. In order to detect highly expressed genes, fold change analysis was done. Genes with 1.5 folds up/down-regulation were considered as differentially expressed at a p-value <0.05, Student's t-test. Functional classification of the genes was performed using gene ontology and pathway analysis [15].

### Quantitative RT-PCR of host genes using SYBR Green I

Microarray gene expression data was validated by quantitative RT-PCR as described earlier [15]. The PCR reaction was performed in triplicates using ABI 7300 real-time PCR system (Applied Biosystems, Foster City, CA, USA) with Quanti Tect SYBR green RT-PCR kit (Qiagen, Germany). Reaction efficiency was calculated by using serial 10-fold dilutions of the housekeeping gene- *β*-actin and the sample genes. Melting curve analysis was performed to verify product specificity. All quantitations (threshold cycle [CT] values) were normalized to that of *β*-actin to generate ΔCT, and the difference among the ΔCT value of the sample and that of the reference (uninfected sample) was calculated as -ΔΔCT. The relative level of gene expression was expressed as 2^-ΔΔCT. ^Primer sequences for the genes of interest were obtained from primer bank and also designed using Primer Express (Applied Biosystems). The primer sequences used in this study are as follows:

*Beta-Actin_F *5'- CATGAAGTGTGACGTGGACATCC-3'; *Beta-Actin_R *5'-GCTGATCCACATCTGCTGGAAGG-3'; *TNFRSF1A_F *5'-TTGCATCCTAGCCCAGCAG-3'; *TNFRSF1A_R *5'-CTGACCCTGGAAAGAAAAGTC-3'; *IL8_F *5'-TGCCAAGGAGTGCTAAAG-3'; *IL8_R *5'-CTCCACAACCCTCTGCAC-3'*;B2 M *(*Beta-2-microglobin*)*_F *5'-ATGTCTCGCTCCGTGGCCTTA-3'; *B2 M *(*Beta-2-microglobin*)*_R *5'-ATCTTGGGCTGTGACAAAGTC-3'; *STAT1_F *5'- CCATCCTTTGGTACAACATGC-3'; *STAT1_R *5'-TGCACATGGTGGAGTCAGG-3'; *IFNβ_F *5'-CAGCAATTTTCAGTGTCAGAAGC-3'; *IFNβ_R *5'-TCATCCTGTCCTTGAGGCAGT-3'.

### Western Blot analysis

Total cellular protein from control and infected A549 cells at different time points post infection were isolated using RIPA lysis buffer. Equal amount of proteins (10 μg) from cell extracts were separated by 12.5% SDS-polyacrylamide gel electrophoresis (12.5% SDS-PAGE) and transferred to Hybond-C (Amersham Biosciences) membrane with an electrotransfer apparatus (Cleaver Scientific Ltd) at 10 Volts (100 mA) for 1 h 30 min. Primary and secondary antibody interaction was performed in phosphate-buffered saline (pH 7.5). Primary antibodies used were rabbit anti-STAT1, mouse anti-CASP3 and mouse anti-*β*-actin antibody (Santa Cruz Biotechnology, Inc. Santa Cruz, CA. USA). The secondary antibodies were mouse anti-rabbit and goat anti-mouse secondary antibodies (Santa Cruz Biotechnology, Inc. Santa Cruz, CA. USA) labeled with Horseradish peroxidase (HRP). The protein bands were developed with 3, 3'-Diaminobenzidine tetrahydrochloride (DABT) and Hydrogen peroxide (H_2_O_2_) staining.

## Results

### Host gene expression profile in response to pandemic (H1N1) 2009 virus infection

**Figure 1:**
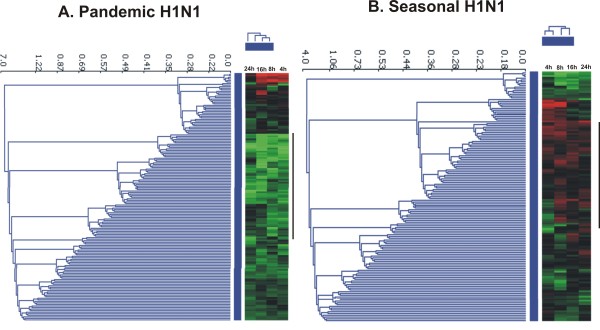
**Hierarchical clustering of differentially expressed genes in A) pandemic influenza H1N1 and B) seasonal influenza virus infected A549 cells at different post-infection time points**. Expression of genes with fold change >+/- 1.5 and p < 0.05 were considered as differentially expressed. Data presented are averaged gene expression changes for 2 different replicates. Black bar indicate expression pattern of immune genes in the two virus infected cells.

The gene expression profile to pH1N1 infection was studied at 4 different time points post infection in order to understand the host responses at different stages of virus infection. Figure 1A shows overall gene expression profile in response to pH1N1 infection. The genes showing increased expression compared to controls at all the time points were mainly involved in T-cell activation and proliferation and enzyme linked protein signaling whereas, genes showing decrease in expression were mostly involved in regulation of apoptosis and NF-κB mediated signaling (Table 1A).

**Table 1:** Significantly enriched Gene Ontology terms in response to infection with pandemic H1N1(2009) and seasonal H1N1 influenza viruses

A. **Pandemic H1N1**			A. **Seasonal H1N1**		
**Term**	**%**	**P-Value**	**Term**	**%**	**P-Value**

regulation of cell proliferation	30.2	2.70E-13	regulation of apoptosis	27	1.80E-17
regulation of apoptosis	28.1	2.00E-11	regulation of cell proliferation	20.9	1.60E-10
leukocyte activation	15.6	1.20E-09	positive regulation of apoptosis	14.7	1.50E-09
response to cytokine stimulus	10.4	3.50E-09	regulation of cell cycle	12.9	2.40E-09
regulation of protein kinase activity	15.6	1.00E-07	negative regulation of apoptosis	12.9	7.60E-09
lymphocyte activation and immune response	12.5	1.30E-07	protein amino acid phosphorylation	17.2	2.00E-08
cytokine-mediated signaling pathway	8.3	5.20E-07	lymphocyte activation	8.6	7.20E-07
positive regulation of signal transduction	12.5	6.10E-06	immune system development	9.8	1.00E-06
positive regulation of transcription from RNA polymerase II promoter	13.5	9.60E-06	transmembrane receptor protein tyrosine kinase signaling pathway	8	1.50E-05
inflammatory response	11.5	8.40E-05	regulation of DNA replication	4.3	9.20E-05
regulation of I-kappaB kinase/NF-kappaB cascade	7.3	9.90E-05	positive regulation of B cell activation	2.5	6.90E-03
MAPKKK cascade	8.3	2.90E-04	JAK-STAT cascade	2.5	1.10E-02

At the early stages of virus infection i.e. at 4 hpi, we found up-regulation of immune genes like TNF-*α*3, EGR-1, IL6R, v-FOS, v-JUN. However, IL13RA, IL3RA, IL4, STAT1, STAT4 were down-regulated at this time point of infection. At 8 hpi there was further increase in the expression of EGR-1, IL6R, TNF-*α*3, v-FOS and v-JUN genes and up-regulation of other immune responsive genes like IL-8. Surprisingly, we observed more number of immune genes getting down-regulated at this stage as compared to 4 hpi. The down-regulation of this set of immune genes was more prominent at later stages (16 and 24 hpi) of infection with pandemic H1N1. Genes involved in intracellular signaling and DNA repair like Topoisomerase II, MAP2K6 were also found to be down-regulated at 8 hpi. Higher expression of IL8, TNF-*α*3, TNFR-6, CXCR4, EGR-1, v-JUN was found at 16 hpi. Gene coding for IL13RA showed continued down-regulation at this stage of infection. Interestingly, at 24 h post infection there was no further increase in immune responsive genes but down-regulation of STAT4, TNFR, IL4R, and TNF6 genes were found.

### Host gene expression profile in response to seasonal H1N1 virus infection

In case of seasonal H1N1 infection there was up-regulation of very few immune responsive genes at the early stage (4 hpi) of infection. In fact, there was down-regulation of genes involved in innate immune response like IL2, IL15 and STAT1 at early stages (4 and 8 hpi) of infection. Figure 1B shows hierarchical clustering of overall cellular gene expression profile in response to seasonal H1N1 virus infection in A549 cells. Gene ontology analysis of differentially expressed genes at all the time points showed not much differences in the type and functions of host genes affected by the pH1N1 and seasonal influenza virus infections (Table 1A and Table 1B). The total number of differentially expressed genes at different post infection time points during the two virus infections is given in Table 2.

**Table 2:** Summary of genes differentially expressed in response to infection with pandemic and seasonal H1N1 viruses

Time-points post infection	Genes qualifying quality criteria in replicated experiments	Differentially expressed genes (+/- 1.5 folds, p < 0.05)	Up-regulated genes	Down-regulated genes
**Seasonal H1N1**				

4 h	230	60	28	32

8 h	147	44	28	16

16 h	164	63	30	33

24 h	212	102	51	51

**Pandemic H1N1**				

4 h	194	41	8	33

8 h	198	49	14	35

16 h	203	23	13	10

24 h	312	93	28	65

At 4 hour post infection with seasonal H1N1, genes encoding for the ribosomal proteins and DNA modifying enzymes were up-regulated and continued to be up-regulated at all the time points post infection studied in this experiment. Increased level of the expression of immune genes was observed from 16 hpi. Cytokines like CCL5, small inducible cytokine A2, IL8 and other immune responsive genes like STAT1, IRF1 and B2 M were found to be up-regulated at this time point post infection with seasonal influenza virus. At 24 hpi there was further increase in the expression of immune and ribosomal genes. There was up-regulation of additional transcription factors and signaling molecules at 24 hpi. However, some of the cytokines, signaling genes and DNA repair genes were selectively down regulated at all the post infection time points. These genes mainly included IGF2R, Topoisomerase I and IL13RA1. Genes involved in cell cycle like Cyclin G1, G2 were down-regulated at all the time points post infection.

### Comparative analysis of host gene expression responses between pH1N1 and seasonal H1N1 infection

**Figure 2:**
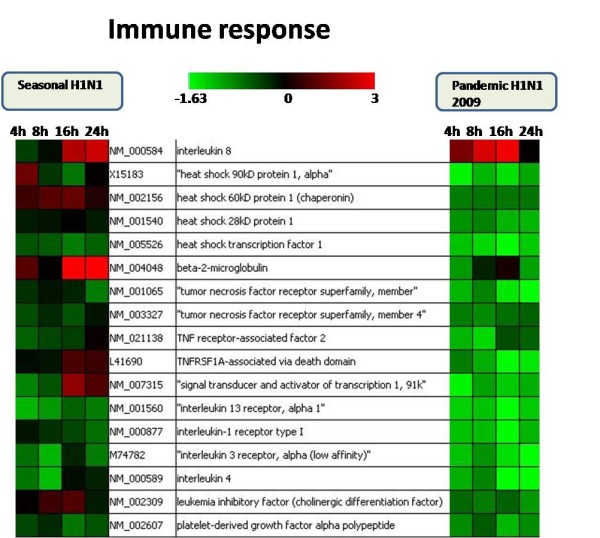
**A comparative analysis of expression profiles of immune responsive genes in response to pandemic H1N1 (2009) and seasonal H1N1 virus infections**.

**Figure 3:**
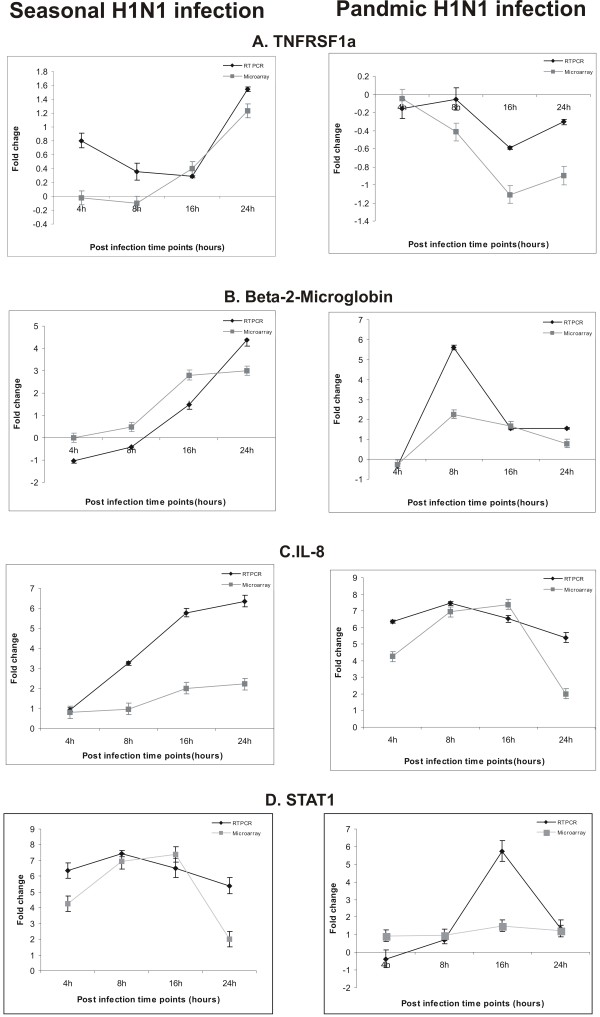
**Validation of microarray data by Real time PCR**. Genes showing differential expression between the two virus infections (seasonal H1N1 and pandemic H1N1) in A549 cells were selectively taken for RT-PCR analysis. The expression of these genes was found to be contrastingly different between the two virus infection in the microarray analysis which correlated with the RT PCR analysis. Error bars indicate mean+/- standard deviation (SD) for 3 replicates. Expression of *β*-actin gene was used as internal control.

**Figure 4:**
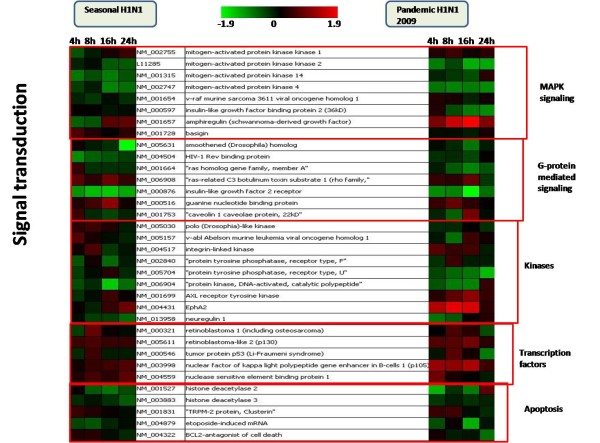
**A comparative analysis of expression profiles of Signal transduction genes in response to pandemic H1N1 (2009) and seasonal H1N1 virus infections**.

Host gene expression profile was compared at all the time points between the two virus infections separately. There were 11, 4, 10 and 20 genes found common between the two virus infections at 4, 8, 16 and 24 hpi respectively. Although there was difference in the level of expression, the expression pattern of the genes was found to be quite similar among the two virus infections. However, the expression of some immune genes like IL4, IL8, B2 M, TNFRF1a and STAT1 was found to be contrastingly different between the seasonal and pandemic influenza virus infection (Figure 2) which was further confirmed using quantitative real-time PCR (Figure 3). The expression of genes involved in immune response, DNA repair and signal transduction were specifically compared between the two infections (pH1N1 and seasonal H1N1 influenza virus) for better understanding of the host responses elicited by them. Analysis revealed that 9 DNA repair genes, 17 immune related genes and 40 genes involved in signal transduction processes were common between the two virus infections (Figure 2 and Figure 4). There was a greater decrease in expression of interleukins, TNFs and heat shock genes in response to pH1N1 infection compared to seasonal H1N1 (Figure 2). There was no significant change in the expression of signaling genes between the two virus infections (Figure 4). However, there was significant decrease in the expression of tyrosine kinases in pH1N1 infected A549 cells compared to seasonal H1N1 infected cells.

### Validation of Microarray data using Real Time PCR and Western blot analysis

**Figure 5:**
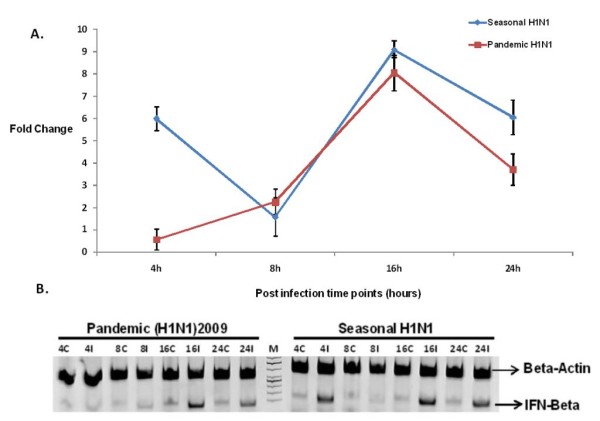
**Expression of IFN-*β *gene in cells infected with seasonal and pandemic H1N1 viruses**. The RNA isolated at different time points post infection (4, 8, 16 and 24 hpi) from both the virus infected cells was used to study gene expression using real-time PCR. The expression values are relative expressions compared to controls. Expression of *β*-actin gene was used as internal control. Error bars indicate mean+/- Standard deviation for 3 replicates. **A**. Expression profile in Real-time PCR experiment. **B**. Visualization of RT-PCR products on 2% Agarose gel. M: 50 bp Marker; 4C-control at 4 hpi; 4I-infected at 4 hpi; 8C-control at 8 hpi; 8I-infected at 8 hpi; 16C-control at 16 hpi; 16I-infected at 16 hpi; 24C-control at 24 hpi; 24I-infected at 24 hpi.

**Figure 6:**
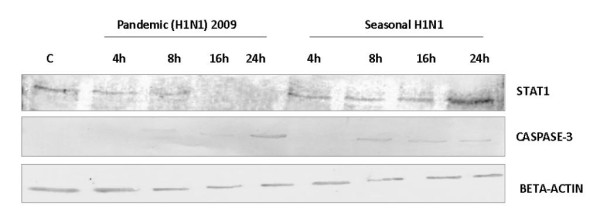
**STAT1 and Caspase 3 protein expression in A549 cells infected with pandemic H1N1 and seasonal H1N1 viruses**. The expression of proteins was studied by Western blot analysis. Equal amount of protein (10 μg) from infected A549 cells with the two viruses (pandemic H1N1 and seasonal H1N1) at different post infection time points (4, 8, 16 and 24 hpi) was transferred to nitrocellulose membrane. After treating with respective anti-protein antibodies the membranes were developed using 3, 3'-Diaminobenzidine tetrahydrochloride (DABT) and hydrogen peroxide (H_2_O_2_) staining. Lane C: Control A549 cells. Expression of *β*-actin was used as internal control.

Expression of selected genes namely STAT1, B2 M, TNFRSF1A and IL8 known to be involved in immune response to influenza A virus infection was validated using Real-time PCR, which correlated with the microarray results (Figure 3). Expression of IFN-*β*, an important antiviral factor was studied using Real time PCR (Figure 5). STAT1, which plays a significant role in activation of innate immune response to viral infection, was further studied for protein expression using Western blotting (Figure 6). Expression of apoptotic factor, Caspase 3 (CASP3) was also studied using Western blot analysis and it was found that the pandemic H1N1 virus infection resulted in higher expression of CASP3 in later stages of infection as compared to seasonal H1N1 (Figure 6).

## Discussion

Global spread of pandemic (2009) influenza A (H1N1) virus was found to be much faster as compared to earlier pandemics. Rapid transmission of the pandemic H1N1 virus, its efficiency to infect hundreds of millions of people worldwide and its impact in public health during the peak pandemic period prompted us to initiate study on host gene expression profile to pandemic H1N1 infection in human lung epithelial cells using microarray platform.

In the present study we have compared the host gene expression responses to pandemic H1N1 and seasonal H1N1 virus infection in lung epithelial (A549) cells. Functional analysis of differentially expressed genes in both the infections revealed qualitative similarity in the gene expression profiles i.e. the type of genes affected were found similar between the two virus infections (Table 1A and 1B). However, there was quantitative difference in the number of differentially expressed genes in pH1N1 and seasonal H1N1 infections. The number of differentially expressed genes in response to seasonal H1N1 infection was higher than that of the pH1N1 at all the time points. This indicates that higher numbers of host genes were getting influenced by seasonal H1N1 than the pandemic H1N1 virus infection. There was a difference in the infection stage at which the genes involved in host immune response were activated during the pandemic and seasonal influenza virus infections. We observed up-regulation of immune genes at early stages of pH1N1 virus infection compared to seasonal H1N1.

However, later stages of infection with pH1N1 were accompanied by a characteristic impairment of the innate immune responses (Figure 2 and Figure 3) characterized by defective cytokine responses as compared to seasonal H1N1. The high infectivity of pandemic H1N1 in animal models as observed in other studies [7,8] could be due to a better subversion of host immune responses by this virus. Cytokine and interferon genes which are important components of innate immunity [16] were also found to be down-regulated at later stages of infection with pH1N1. Similar decrease in immune response during pandemic (H1N1) 2009 virus infection has been reported earlier also [17]. Expression of IFN-*β *gene, which is an important host defense gene, was analyzed by Real-time PCR (Figure 5). IFN-*β *is the most potent antiviral cytokine and is massively produced during Influenza A virus infection [18]. Virus should have capability to overcome antiviral action of interferon during its course of infection in order to replicate efficiently. We found that both the viruses have the property of antagonizing INF-*β*, but pH1N1 infection results in stronger suppression of host INF-*β *expression at early stages (4 hpi) as compared to seasonal H1N1. Also, suppression of INF-*β *was stronger at later stages (24 hpi) of pandemic virus infection. These observations indicate greater replicative ability of pandemic viruses in the host cells as compared to seasonal H1N1 (Figure 5). An early activation of Caspase 3 (Figure 6) is indicative of an early host response to seasonal H1N1 infection as compared to pH1N1. However, at the later stages higher level Caspase 3 expression by pH1N1 infection could be a viral mediated response and the overall Caspase 3 expression pattern indicates a differential ability of the two viruses to induce apoptosis. Probably pH1N1 inhibit apoptosis in the early stages of infection and utilizes cellular machinery more efficiently to get a better replicative ability as compared to seasonal H1N1 virus infection.

To further validate the differences in the host immune responses to seasonal H1N1 and pH1N1 virus infection, we studied the expression of STAT1 protein (Figure 6). STAT1 protein is an important component of Jak-Stat pathway which gets activated at later stages of virus infection. Activation of Jak-Stat pathway results in stimulation of Interferon regulatory genes leading to heightened immune responses [16,18]. We observed higher expression of STAT1 protein in seasonal H1N1 infection at later stages as compared to pH1N1 (Figure 6). This is indicative of suppression of STAT1 at later stages of pandemic H1N1 virus infection, which might decrease the overall host innate immune response. On the other way, suppression of STAT1 leads to inhibition of interferon regulatory genes allowing rapid replication and consequent spread of pH1N1 compared to seasonal H1N1. NS1 protein of influenza virus is a very important component to antagonize host interferon activity to lead virus replication. The pathogenicity and transmission of influenza A viruses are likely determined in part by replication efficiency in human cells, which is the net effect of complex virus-host interactions. A recent study have shown that the influenza A virus that circulate in human differ markedly in the ability of their NS1 protein to block the activation of interferon regulatory gene (IRF3) and interferon beta transcription [19]. Probably NS1 protein of pH1N1 is more potent in antagonizing host interferon activity compared to seasonal H1N1 viruses in mammalian cell line (A549) which facilitates rapid viral replication.

Indian isolates of 2009 pandemic H1N1, although overall similar to Mexican strain is reported to cause greater mortality in India on the basis of clinical records. During the peak pandemic period almost 90% cases were found positive for pH1N1 in New York City and other many other regions. However, both seasonal-H1N1 and pandemic H1N1 were represented in almost equal proportion in Indian population during the peak pandemic period [20]. Although many gaps remain in understanding how a pandemic influenza virus behaves, spread and affect the community, in Indian scenario, along with viral factors other socio-economic and environmental factors may also be involved in high pathogenesis of pandemic H1N1 virus.

## Conclusion

It is well established that pH1N1 is highly transmissible as it has rapidly spread to large number of countries within a very short period of time. We propose that high transmissibility of pandemic H1N1 virus in the year 2009-2010 is because of its better subversion of host immune responses compared to the seasonal influenza viruses. Additionally, lack of earlier immunity to pH1N1 virus makes it more pathogenic as compared to seasonal H1N1.

## Abbreviations

pH1N1: (pandemic H1N1); hpi: (hours post infection); aRNA: (amino allyl amplified RNA); GO: (gene ontology)

## Competing interests

The authors declare that they have no competing interests.

## Authors' contributions

AKC and ACM conceived the idea and initiated the project. AKC contributed to project design and supervised the project. SM, AKC, VCV and SDP performed the experiments. AKC, SM and VCV performed data analysis and bioinformatics studies. AKC, SM and ACM wrote the paper. All authors read and approved the final manuscript.
